# Rivaroxaban’s vascular dose for the neurovascular clinician

**DOI:** 10.1186/s41016-024-00373-4

**Published:** 2024-07-03

**Authors:** Ji Y. Chong, Kalimullah Jan

**Affiliations:** 1 Stroke Center, New York Medical College, Westchester Medical Center, Valhalla, NY USA; 2 Neurovascular Fellow, New York Medical College, Westchester Medical Center, Valhalla, NY USA

**Keywords:** Stroke, Atherosclerosis, Rivaroxaban, Cardiovascular, Cerebrovascular

## Abstract

Rivaroxaban, a direct oral anticoagulant, has proven efficacy and safety at its standard dose in the treatment and prevention of various vascular conditions. These include the treatment of venous thromboembolism and stroke prevention in non-valvular atrial fibrillation. A “very low” vascular dose of rivaroxaban, when combined with low-dose aspirin, has been demonstrated to reduce major adverse cardiovascular events, including stroke, in both acute and chronic coronary syndrome. The combination of rivaroxaban and low-dose aspirin could potentially offer an additional strategy for stroke prevention in selected non-atrial fibrillation patients who are at a high risk of stroke.

Over the last decade, rivaroxaban, a direct oral anticoagulant, has emerged as a promising treatment option for various cerebrovascular conditions. Rivaroxaban reversibly binds to the active site on factor Xa in the coagulation cascade, thereby inhibiting thrombin and fibrin formation. It has a serum half-life of 5–9 h in healthy subjects but in older patients, the half-life can be about 13 h [1]. At its full therapeutic dose (i.e., 20 mg daily for normal renal function and 15 mg daily for renal impairment, i.e., estimated glomerular filtration rate between 15 and 30 ml/min), it has been shown to reduce the risk of ischemic stroke in patients with non-valvular atrial fibrillation, as evidenced by the ROCKET AF trial. When compared to warfarin, it has a relatively safe profile [2]. The same dose is applicable in treating venous thromboembolism (VTE) but only after a 3-week course of 15 mg twice daily rivaroxaban dosing without a heparin lead-in phase, as per EINSTEIN-DVT and EINSTEIN-PE trials data [3]. Moreover, the half-dose of rivaroxaban (i.e., 10 mg daily) has been proven to reduce recurrent VTE in patients who have completed 6–12 months of VTE treatment and are at equipoise for continued anticoagulation (EINSTEIN-CHOICE trial) [4].

The ATLAS TIMI 46 trial was a dose-finding study that identified the optimal balance of antithrombotic benefit and bleeding risk at a dose of 2.5 mg twice daily [5]. From a cardiovascular or neurovascular physician’s perspective, the use of rivaroxaban, particularly the “very low” vascular dose of 2.5 mg twice daily, presents both opportunities and challenges. The ATLAS ACS 2-TIMI 51 trial established that both the low dose (5 mg twice daily) and the “very low dose” (2.5 mg twice daily) of rivaroxaban decreased major adverse cardiovascular events (MACE) in acute coronary syndrome (ACS). While rivaroxaban 5 mg twice daily increased the risk of bleeding and intracranial hemorrhage (ICH), the 2.5 mg twice daily dose had a relatively lower bleeding risk compared to the 5 mg twice daily dose (0.1% vs 0.4%, *P* = 0.04) and resulted in significantly less mortality from cardiovascular causes [6]. Unfortunately, these pioneering results did not lead to a change in clinical practice, even though the European Society of Cardiology endorsed this 2.5 mg BID dosing regimen for ACS patients. Recently, a short-term, low dose of rivaroxaban (i.e., 5 mg twice daily) was found to be non-inferior to the traditional parenteral anticoagulant, noxaparin, in ACS (H-REPLACE trial) [7].

In patients who enter the chronic coronary syndrome (CCS) window, that is, one year after the index ACS or percutaneous coronary intervention, aspirin is still the cornerstone of therapy despite its suboptimal effect in reducing cardiovascular risk. The COMPASS trial examined the potential benefits of using a low dose of rivaroxaban instead of aspirin, and very low doses of rivaroxaban in combination with aspirin in cases of CCS. The trial, which included 27,000 non-atrial fibrillation patients, demonstrated that a combination of very low-dose rivaroxaban (i.e.; 2.5 mg twice daily) and aspirin reduced a composite outcome of cardiovascular death, stroke, or myocardial infarction by 24% and overall mortality by 18% compared with aspirin alone. Major bleeding occurred in 3.1% of the combination group vs 2.8% in the rivaroxaban 5 mg twice daily monotherapy group vs 1.9% in the aspirin monotherapy group. Although this combined regimen did increase the risk of major bleeding, it didn’t increase the risk of ICH or fatal bleeding. Thus, the net clinical benefits for secondary cardiovascular prevention were not undermined, particularly in patients with high baseline cardiovascular risk [8]. Additionally, the VOYAGER-PAD trial further reinforced these findings in patients with peripheral artery disease (PAD) who were undergoing revascularization. As such, the Food and Drug Administration in 2018 added a new indication for rivaroxaban 2.5 mg twice daily with aspirin to reduce MACE (cardiovascular death, myocardial infarction, and stroke) in patients with coronary artery disease and PAD, also endorsed by American College of Cardiology/American Heart Association with a class 2a recommendation.

Currently, no randomized controlled trial (RCT) has specifically investigated the effectiveness of rivaroxaban 2.5 mg BID in combination with low-dose aspirin for the prevention of ischemic stroke in high-risk patients without atrial fibrillation, such as those with Intracranial Atherosclerotic Disease (ICAD) or symptomatic carotid disease. Nonetheless, the COMPASS trial included patients with asymptomatic carotid artery stenosis of 50% or more or those who had previously undergone carotid revascularization [9]. A comprehensive sub-analysis of the COMPASS trial demonstrated that compared to aspirin monotherapy, the combination of rivaroxaban 2.5 mg BID and aspirin significantly reduced the overall incidence of stroke, particularly ischemic/uncertain stroke, which was reduced by nearly 50% (0.7% per year versus 1.4% per year) without significantly increasing hemorrhagic stroke [10]. For the Neurovascular physician, these findings are potentially practice-changing, but one should bear in mind that patients with recent strokes were excluded from this trial. There are ongoing RCTs targeting this population as well. For instance, the CATIS-ICAD trial (NCT04142125) is exploring the safety and efficacy of low-dose rivaroxaban plus aspirin vs aspirin with recent ischemic stroke or transient ischemic attack due to ICAD. Another ongoing trial in China, AA-ICAS, (NCT05700266), aims to compare DAPT (aspirin with clopidogrel) with rivaroxaban 2.5 mg BID with aspirin 100 mg for secondary stroke prevention in ICAD-related stroke.

In summary, the use of “vascular dose” rivaroxaban 2.5 mg twice daily in combination with low-dose aspirin could potentially provide neurovascular practitioners with an additional approach for stroke prevention in select non-atrial fibrillation patients who are at high risk for stroke.

## Data Availability

Not applicable.

## Abbreviations

ACS: Acute coronary syndrome
BID: Twice daily
CCS: Chronic coronary syndrome
DAPT: Dual antiplatelet therapy
ICAD: Intracranial atherosclerotic disease
ICH: Intracranial hemorrhage
MACE: Major adverse cardiovascular events
PAD: Peripheral artery disease
RCT: Randomized controlled trial
VTE: Venous thromboembolism
